# Strategic modulation of response inhibition in task-switching

**DOI:** 10.3389/fpsyg.2013.00545

**Published:** 2013-08-22

**Authors:** Kai Robin Grzyb, Ronald Hübner

**Affiliations:** Department of Psychology, Universität KonstanzKonstanz, Germany

**Keywords:** response repetition, response inhibition, task switching, strategic processing, response conflict

## Abstract

Residual activations from previous task performance usually prime the system toward response repetition. However, when the task switches, the repetition of a response (RR) produces longer reaction times and higher error rates. Some researchers assumed that these RR costs reflect strategic inhibition of just executed responses and that this serves for preventing perseveration errors. We investigated whether the basic level of response inhibition is adapted to the overall risk of response perseveration. In a series of 3 experiments, we presented different proportions of stimuli that carry either a high or a low risk of perseveration. Additionally, the discriminability of high- and low-risk stimuli was varied. The results indicate that individuals apply several processing and control strategies, depending on the mixture of stimulus types. When discriminability was high, control was adapted on a trial-by trial basis, which presumably reduces mental effort (Experiment 1). When trial-based strategies were prevented, RR costs for low-risk stimuli varied with the overall proportion of high-risk stimuli (Experiments 2 and 3), indicating an adaptation of the basic level of response inhibition.

## Introduction

The environment is often ambiguous about the appropriate response for a given task. For instance, different features of a stimulus might be associated with different actions, so that stimulus processing activates competing responses, which can result in suboptimal performance or even errors (cf. Desimone and Duncan, 1995). One mechanism to prevent such errors is selective attention that can be used to filter out irrelevant stimulus information (cf. Kahneman and Treisman, 1984; Bundesen, 1990; Hübner et al., 2010). However, in some situations perceptual filtering can be difficult or even impossible (e.g., Stroop, 1935; Simon, 1969; Eriksen and Eriksen, 1974). In these cases suppression of irrelevant response activation might be applied as an alternative mechanism for limiting the error rate (e.g., Ridderinkhof, 2002).

In addition to activation produced by irrelevant features of the current stimulus, residual activation left over from previous task performance can also bias responding. For instance, when participants switch between overlapping tasks that share mental representations, persistent activation of the representations that were involved in performing the previous task, interferes with current task processing, which usually impairs performance (e.g., Allport et al., 1994; Masson et al., 2003; Yeung and Monsell, 2003; see Kiesel et al., 2010, for a review). The interference increases the risk of erroneously re-executing either the previous task (task perseveration errors), or the pervious response (response perseveration error). To control such perseverations, it has been assumed that individuals are equipped with inhibitory mechanisms (e.g., Mayr and Keele, 2000; Hübner and Druey, 2006; Juvina and Taatgen, 2009). The basic idea is that task representations that were active on the previous trial are inhibited—in whole or in part—in order to control the error rate by reducing their perseverative influence on the current processing. From the different components of a task representation that could be inhibited, the current study is concerned with the inhibition of response representations (Hübner and Druey, 2006; Cooper and Marí-Beffa, 2008). For simplicity, we will call this type of inhibition *response inhibition*.

Given that response inhibition is an anti-perseverative mechanism in task switching, an important question is how flexibly its strength can be adjusted to the risk of response perseveration which is related to the degree of irrelevant response activation. For instance, stronger inhibition seems advantageous in task contexts where irrelevant stimulus features frequently reactivate the previous (but now wrong) response. This would increase the overall risk of perseveration, compared to conditions, where such activations occur less frequently. Thus, a reasonable hypothesis is to assume that the strength of response inhibition is strategically adjusted to the overall risk of response perseveration errors (Hübner and Druey, 2006; Steinhauser et al., 2009). Up to now, however, evidence for this strategic-adaptation hypothesis is inconclusive (Grzyb and Hübner, 2013a). In typical task-switching studies investigating the adaptability of response inhibition, the ratio of high-risk to low-risk trials is manipulated, i.e., the proportion of trials with a stimulus that increases the risk of response perseveration is varied. If the strategic-adaptation hypothesis is correct, then response inhibition should increase with the proportion of high-risk stimuli. However, in a previous study (Grzyb and Hübner, 2013a) no proportion effect was found. Yet, to conclude that there is no strategic adaptation might be premature, because in that study high-risk stimuli could easily be discriminated from low-risk stimuli perceptually. As a consequence, participants could have adjusted response inhibition to the current stimulus-type. If such a specific processing of different stimulus-types is applicable on a trial-by-trial basis, then an overall strategic adaptation of response inhibition to the proportion of high-risk stimuli might be unnecessary.

Therefore, the aim of the present study was to investigate how trial-based strategies affect the overall adaptation of response inhibition. As our results show, strategic adaptation to overall control demands takes place only when trial-based strategies are prevented. But before we report our results in detail, we review the relevant literature on response inhibition in task-switching studies.

### Response inhibition in task-switching

In task-witching studies a characteristic interaction can be observed between the transition of tasks and responses (e.g., Rogers and Monsell, 1995; Kleinsorge and Heuer, 1999; Meiran, 2000; Meiran et al., 2000; Schuch and Koch, 2004; Hübner and Druey, 2006, 2008; Cooper and Marí-Beffa, 2008; Druey and Hübner, 2008a; Koch et al., 2011). When comparing performance on trials where the response of the previous trial repeats with performance on trials where the response shifts (RSs), response repetition (RR) benefits can be found on task-repetition trials and RR costs on task-switch trials. Several ideas have been proposed for explaining this interaction (e.g., Rogers and Monsell, 1995). Here we focus on the idea that responses are inhibited after their execution to prevent perseveration errors.

The idea of response inhibition as an anti-perseverative mechanism has a long tradition (e.g., Smith, 1968), but recently gained additional attention in the area of task switching. Cooper and Marí-Beffa (Cooper and Marí-Beffa, 2008; Marí-Beffa et al., 2012), for instance, argued that in natural contexts a switch from one task to another is normally accompanied by a shift from one response or effector to another (see also, Mayr and Bryck, 2007). In these cases, inhibiting a response after its execution would facilitate a switch from one action to another by inducing a RS bias. In task-switching studies, however, response mappings often overlap between tasks such that the same response is also part of different tasks (e.g., judging the parity of numerals by pressing one of two response keys, and categorizing letters as consonants or vowels by pressing the same keys). With such stimulus-response mappings, the response can repeat even if the task switches. As a result, RR usually leads to performance costs, presumably because the inhibition has to be overcome to re-execute the previous response (Hübner and Druey, 2006). The situation is different on task-repetition trials. Here, RR occurs together with a repetition of the stimulus category (cf. Pashler and Baylis, 1991), so that episodes of previous and current trial features match (Altmann, 2011). The corresponding positive effects usually outweigh the negative effect of response inhibition (but see, e.g., Cooper and Marí-Beffa, 2008). In sum, RR produces benefits on task-repetition trials, but costs on task-switch trials, which explains the observed interaction between the transition of tasks and responses in task-switch studies.

### Strategic adaptation of response inhibition

If inhibition is considered as control mechanisms, then an important question is whether its strength can be modulated strategically. For the Simon task, for instance, where response inhibition also plays an important role for control, it has been shown that the strength of inhibition can strategically be adapted to different demands, but only when sufficient information about the corresponding condition is provided (Hübner and Mishra, 2013). Note that such a strategic adaptation must not necessarily be based on a deliberate choice of a certain strength of response inhibition. It is also conceivable that the strength results from a more abstract feed-back loop that simply controls the error rate. The specific mechanisms might remain unconscious. Here, we simply mean by “strategy” any top-down influence on performance that depends on the conditions of the specific task context. In task switching, for instance, the inhibition of a just abandoned task (*backward inhibition*; Mayr and Keele, 2000) is assumed to be stronger in blocks where tasks always switch compared to blocks were the frequency of task switches is lower (e.g., Dreisbach and Haider, 2006; Philipp and Koch, 2006). This inhibition seems to be adaptive, because frequent task switches increase the interference between tasks, increasing the difficulty of task performance. This means that the risk of an erroneous re-execution of the just performed task (task perseveration error) is increased, which would be counteracted by stronger backward inhibition. Similarly, it has been hypothesized that the strength of response inhibition is strategically adapted to the risk of an erroneous re-execution of the last response (response perseveration error; Hübner and Druey, 2006). The risk should be especially high if stimulus features frequently activate the previous but now wrong response.

Unfortunately, evidence for a strategic adaptation of response inhibition in task switching is inconclusive. Studies supporting the strategic-adaptation hypothesis usually compared RR effects between low- and high-risk task-switching contexts (e.g., Lien et al., 2003; Hübner and Druey, 2006). In a study by Hübner and Druey (2006), for instance, univalent and bivalent stimuli served as low- and high-risk stimuli, respectively (a description of univalent and bivalent stimuli can be found in Table 1). The risk of perseveration is low for univalent stimuli, because they activate only the relevant task and the correct response. Bivalent stimuli, in contrast, activate both tasks and, thus, also a stimulus category and an associated response of the irrelevant task. Accordingly, Hübner and Druey (2006) reasoned that the latter stimuli should pose a higher risk of response perseveration error than univalent stimuli. Consequently, if the proportion of bivalent stimuli is increased response inhibition should strategically be increased in order to control response perseverations. Stronger inhibition, however, should also increase the costs (or reduce the benefits) if a response has to be repeated. Indeed, in line with this reasoning, Hübner and Druey (2006) observed larger RR costs on task-switch trials and smaller RR benefits on task-repetition trials in conditions with 100% high-risk stimuli, compared to conditions with 100% low-risk stimuli.

**Table 1:** **Categorization of the applied stimulus-types with respect to their item congruency and valency**.

**Valency**	**Item-congruency**		
	**Neutral**	**Congruent**	**Incongruent**
Univalent	Neutral (e.g., *G* or *6*)	*Univalent*-congruent (e.g., KGK or 868)	*Univalent*-incongruent (e.g., AGA or 363)
Bivalent	–	*Bivalent*-congruent (e.g., 8G8 or K6K)	*Bivalent*-incongruent (e.g., 3G3 or A6A)

Note. The item-congruency feature specifies whether a category and its corresponding response are associated with the task-irrelevant stimulus item and if so, how this response is related to the correct response (none = neutral; same as correct response = congruent; different than correct response = incongruent). The valency feature specifies how many tasks can be performed with a stimulus (one = univalent; two = bivalent). The tasks in the experiments were consonant/vowel judgments of letters and even/odd judgments of numbers indicated by left/right button presses. Examples of the stimulus-types assume that the target item (G or 6) is located in the middle of three-item stimulus. In the experiments, however, target items were presented randomly either in the central or the outer locations of the stimulus array on Task 2.

A recent study where different proportions of high-risk stimuli were used (Grzyb and Hübner, 2013a), however, questions whether Hübner and Druey's (2006) findings can best be explained by a strategic adaptation of response inhibition. In that study Grzyb and Hübner used bivalent-incongruent stimuli as high-risk stimuli (see Table 1). These stimuli pose a rather high risk of response perseveration, because they not only activate the wrong task (due to bivalency) but also the wrong response (due to incongruency). Therefore, on a RS trial, the activation of the wrong response adds to the activation carried over from the previous trial thereby increasing the risk of an erroneous RR. For comparison, univalent stimuli served as low-risk stimuli. Replicating the results of Hübner and Druey (2006), Grzyb and Hübner (2013a) found larger RR costs in conditions with 100% high-risk stimuli than in conditions with 100% low-risk stimuli. Unexpectedly, however, RR costs for the respective stimulus-types remained the same when the stimulus types were mixed (50% low-risk, 50% high-risk) within a block of trials. This trial-based variation in RR costs cannot be explained by an overall response-inhibition strategy that depends on the proportion of the stimulus types. Rather, the result suggests that some trial-based mechanisms—related to the current stimulus type—modulated the RR costs.

To explain the stimulus-type dependent RR costs, Grzyb and Hübner (2013a) proposed the *amplification of response conflict* (ARC) account. According to this idea, RR costs do not only vary with the strength of response inhibition, but also with the current stimulus type. Given a certain strength of response inhibition, different RR costs result for high and low-risk stimuli, because response inhibition modulates response conflict differently depending on the overlap between the inhibited response and the correct response. On RR trials, for instance, the inhibited and the correct response fully overlap. Thus, for a bivalent-incongruent stimulus the response conflict on RR trials is amplified, because the correct response is inhibited, while the activation of the competing wrong response remains unaffected. On RS trials, in contrast, the response conflict is smaller, because response inhibition now exclusively reduces the activation of the wrong response. Note that these effects are *not* the consequences of varying degrees of response inhibition. Nonetheless, this pattern of effects results in larger RR costs for bivalent-incongruent stimuli, compared to low-risk (e.g., neutral) stimuli, which do not elicit a response conflict (for the effect of ARC on RR benefits on task-repetition trials see Grzyb and Hübner, 2013b).

Do the results of Grzyb and Hübner (2013a) imply that there is no strategic adaptation of response inhibition? Such a conclusion might be premature. One reason is that Grzyb and Hübner mixed only neutral (e.g., “#A#”) and bivalent-incongruent (e.g., “3A3”) stimuli. Because these two stimulus types can be easily discriminated perceptually, participants might have applied a stimulus-type specific inhibition strategy in a trial-by-trial manner, especially, as bivalency was perfectly coupled with response conflict (Koch et al., 2010). As a consequence, an overall strategy might not have been necessary. Moreover, because such a stimulus-type specific response inhibition and ARC would affect the size of RR costs similarly, Grzyb and Hübner's (2013a) trial-based effect might have, at least partially, be the result of stimulus-type specific response inhibition and not only of ARC.

### Objective of the current study

**Figure 1:**
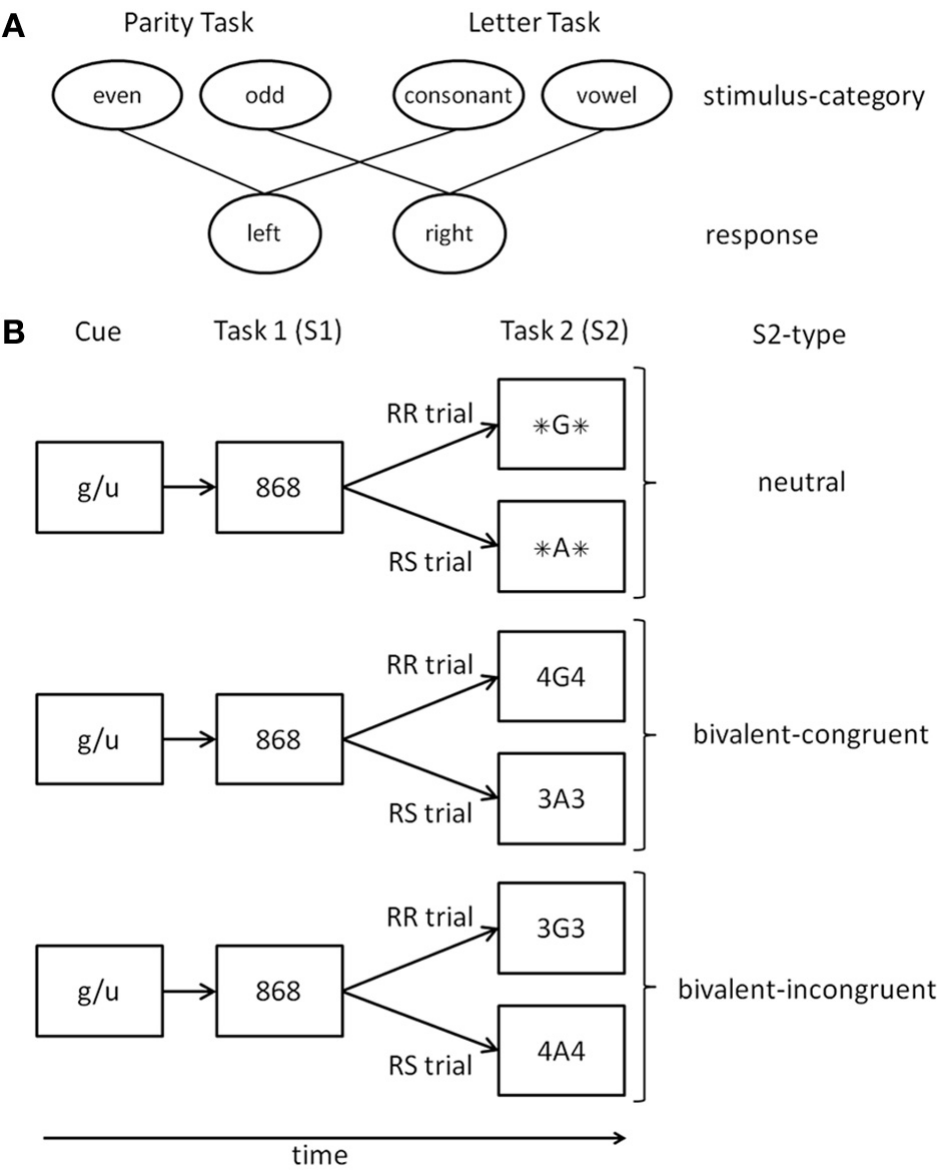
**(A)** Mapping of stimulus categories to responses for the two tasks. **(B)** Schematic examples of trials in different conditions. A cue indicates the relevant judgment for Task 1. Task 2 is always a switch to the alternative judgment. In the depicted example Task 1 is the even-odd judgment (the cue “g/u” is an abbreviation of the German category words “gerade” (even) and “ungerade” (odd)). RR, response repetition; RS, response shift; For details see text.

The aim of the present study was to again test the idea that response inhibition can strategically be adapted to the overall risk of response perseveration. This time, however, we tried to prevent trial-based strategies by including a further stimulus type that makes perceptual discriminability rather difficult. As in Grzyb and Hübner (2013a), we used two-task sequences in which a task-switch was required on every trial (cf. Figure 1). To control for effects of previous-trial congruency on RR costs (cf. Druey and Hübner, 2008b; Grzyb and Hübner, 2012), we kept the stimulus type for the first task constant, and only varied the type for the second task. For both tasks compound stimuli were used, consisting of a target item and a distractor item (see Table 1). The strength of response inhibition was assessed by the RR costs for responses to stimuli in the second task.

In a first step, we tested the effect of perceptual discriminability on RR costs. Therefore, in Experiment 1, we replicated the results of Grzyb and Hübner (2013a) with an even lower proportion of high-risk stimuli. Then, in Experiment 2, we decreased the perceptual discriminability between high- and low-risk stimuli by uncoupling bivalency and incongruency. This was obtained by including bivalent-congruent stimuli in the second task. As a result, trial-based effects were indeed reduced. Finally, in Experiment 3, we tested the strategic-adaptation hypothesis by mixing the same three stimulus types as in Experiment 2, but by further reducing the proportion of high-risk stimuli. The results clearly show that the overall strength of response inhibition can be gradually adapted to the proportion of high-risk stimuli.

## Experiment 1

Experiment 1 should replicate the main results of Grzyb and Hübner (2013a), i.e., larger RR costs for bivalent-incongruent stimuli than for neutral ones, and provide a baseline for Experiment 2. Whereas in Grzyb and Hübner (2013a) the proportion of bivalent-incongruent stimuli was 1/2, it was reduced to 1/3 in the present experiment. Nonetheless we expected the same pattern of RR costs as in Grzyb and Hübner (2013a). According to the ARC account, RR costs for bivalent-incongruent stimuli should be increased, because response inhibition amplifies the response conflict elicited by these stimuli only on RR trials. Moreover, because bivalency was easily discriminable and uniquely coupled with incongruency, it was again possible to use a stimulus-type specific response inhibition. If such a strategy would indeed be applied, it would also increase RR costs specifically for bivalent-incongruent stimuli.

### Method

#### Participants

Thirty-four students of the Universität Konstanz participated in the experiment (6 male; *M* = 22 years). All participants had normal or corrected-to-normal vision and were either paid 8 Euro per hour or fulfilled a course requirement.

#### Apparatus and stimuli

The stimuli were presented on a 19-inch color monitor with a resolution of 1280 × 1024 pixels and a refresh rate of 60 Hz. A PC controlled stimulus presentation and response registration running the software package Presentation (Neurobehavioral Systems, Albany, CA, USA; www.neurobs.com). The two buttons of a regular computer mouse served as response buttons. The stimuli were constructed using letters (G, K, R, A, E, U) and numerals (4, 6, 8, 3, 5, 7) as stimulus items. There were also three neutral symbol (^*^, &, %) that were unrelated to any task. Each stimulus array—S1 for Task1, S2 for Task2—consisted of three items. Similar to a flanker stimulus, two identical items were presented to both sides of a central item. For S1 the target item was always the central item. For S2, on each trial it was randomly determined whether the central item or the flanker items were the target. The spatial uncertainty of the target item should allow for a strong effect of the distractor item which should increase bivalency and incongruency effects. The items in S1 were always univalent-congruent, i.e., target and distractor items were related to the same task (letters *or* numerals) and were associated with the same response (cf. Table 1). S2 was either neutral or bivalent-incongruent. Neutral stimuli were composed of the target item and a neutral symbol as distractor items. Bivalent-incongruent stimuli consisted, i.e., target and distractor items were related to different tasks (a letter *and* a numeral) and were associated with different responses. A stimulus pattern subtended a visual angle of approximately 5.5° width and of 2.1° height. The stimuli were displayed in white on a black background.

#### Procedure

At the beginning of each trial a cue was presented for 800 ms that indicated the relevant judgment for Task1 (see Figure 1). Cues were abbreviations of the indicated judgment, i.e., “g/u” (odd/even judgment; German words “gerade” (even) and “ungerade” (odd)) and “k/v” (consonant/vowel judgment; German words “Konsonant” (consonant) and “Vokal” (vowel)). After a blank screen of 200 ms the stimulus S1 for Task1was presented and remained visible until response. The stimulus S2 for Task2 was displayed 1500 ms after S1 or, if the response time for S1 was longer, after that response. The result of a judgment had to be indicated by pressing one of two response buttons (left, right), which were the same for each task. The categories even and consonant were mapped to the left button, odd and vowel to the right button. After an error a short feedback tone (500 Hz, 100 ms) was presented. The next trial started 1000 ms after the second response. Participants were instructed to prepare for the upcoming tasks and to respond as fast as possible while keeping accuracy above 90%. The experiment consisted of 12 blocks each encompassing 72 trials. The first two blocks served as training blocks and were not analyzed.

#### Design

In all experiments the dependent variables were the response latencies to S1 (RT1) and to S2 (RT2) and the corresponding error rates ER1 and ER2. From these measures we calculated RR costs as the mean performance on RR trials minus that on RS trials. The experiment followed a within-participant design with *response transition* (repetition, shift) and *S2 type* (neutral, bivalent-incongruent) as independent variables. Although we included only task-switch trials, due to the two-task sequence procedure, inter-trial sequences were random. Therefore, there could be task repetitions and task shifts from Task 2 on one trial to Task 1 on the next trial. These inter-trial transitions were not analyzed.

### Results

Trials with RT1 > 1500 ms were excluded from the analysis (2.04% of all trials).

#### RT1

The mean latency for correct responses to S1 was 581 ms (*SE* = 18.38 ms).

#### ER1

The mean error rate for responses to S1 was 4.08% (*SE* = 0.0043%).

#### RT2

**Figure 2:**
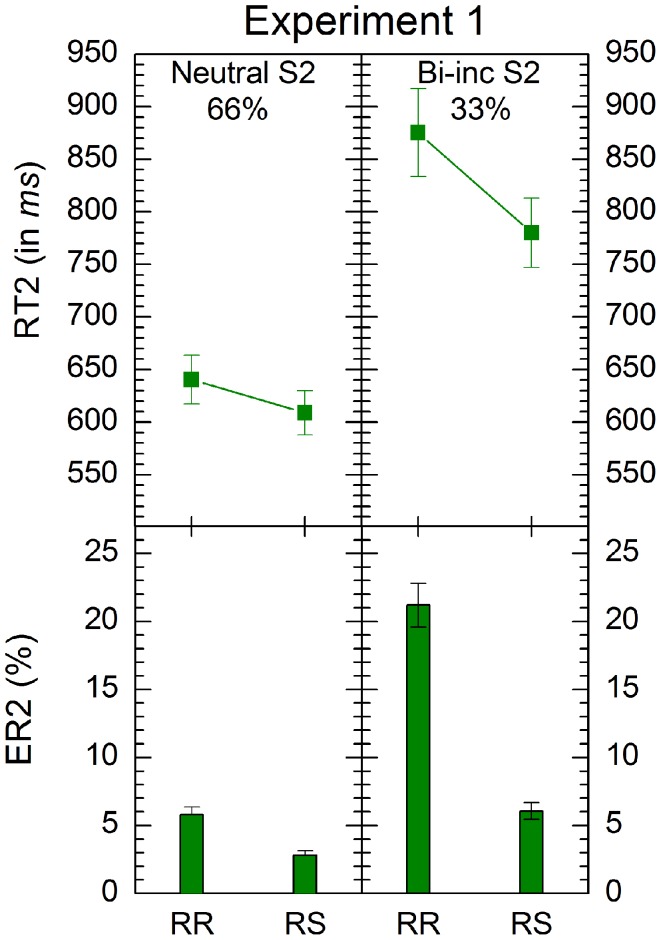
**Mean response times and errors rates in conditions of Experiments 1.** “RR” and “RS” denote response repetition and response shifts, respectively. “Bi-inc S2” denote bivalent-incongruent stimuli on Task2 (see Table 1 for details of stimulus classification). The percentages indicate the relative proportion of the respective stimulus-types. Error bars represent standard errors of the mean.

Anticipatory errors (RT2 < 150 ms) and extreme outliers (RT2 > 3500 ms) were excluded from the analysis of second response (together, less than 0.3% in each condition) as well as trials with incorrect responses to S1. Mean latencies of correct responses were entered into a two-way ANOVA with the independent variables *response transition* (repetition, shift) and *S2 type* (neutral, bivalent-incongruent) realized within participants. Results are depicted in Figure 2.

The analysis revealed significant main effects of *S2 type*, *F*(1, 33) = 109, *p* < 0.001, and *response transition*, *F*(1, 33) = 39.4, *p* < 0.001. Responses to neutral S2 were faster than those to bivalent-incongruent ones (*M* = 625 ms, *SE* = 15.72 ms vs. *M* = 828 ms, *SE* = 27.03 ms) and RRs were slower than RSs (*M* = 758 ms, *SE* = 27.75 ms vs. *M* = 694 ms, *SE* = 22.03 ms). These effect were qualified by a significant interaction between the two variables, *F*(1, 33) = 31.2, *p* < 0.001. RR costs were larger for bivalent-incongruent S2 than for neutral S2 (bivalent-incongruent S2: RR *M* = 875 ms, *SE* = 41.80 ms vs. RS *M* = 780 ms, *SE* = 32.89 ms; neural S2: RR *M* = 640 ms, *SE* = 23.32 ms vs. RS *M* = 609 ms, *SE* = 21.08 ms).

#### ER2

Mean error rates for responses to S2 were subjected to an ANOVA of the same type as for the latencies. The analysis revealed significant main effects of *S2 type*, *F*(1, 33) = 132, *p* < 0.001, and *response transition*, *F*(1, 33) = 74.2, *p* < 0.001. Fewer errors occurred for neutral than for bivalent-incongruent S2 (*M* = 4.30%, *SE* = 0.37%, vs. *M* = 13.6%, *SE* = 1.26%), and RRs produced more errors than RSs (*M* = 13.5%, *SE* = 1.27% vs. *M* = 4.43%, *SE* = 0.41%). The interaction between the two independent variables was also significant, *F*(1, 33) = 60.5, *p* < 0.001. RR costs were larger for bivalent-incongruent S2 than for neutral ones (bivalent-incongruent S2: RR *M* = 21.2%, *SE* = 1.61%, RS *M* = 6.07%, *SE* = 0.62%; neutral S2: RR *M* = 5.80%, *SE* = 0.56%, RS *M* = 2.80%, *SE* = 0.35%).

### Discussion

As expected, we found substantially larger RR costs for bivalent-incongruent stimuli than for neutral ones in both response times and error rates, which replicates and generalizes the findings of Grzyb and Hübner (2013a). It seems that the difference in RR costs between the two stimulus types is independent of their proportion, which is in line with the ARC account (Grzyb and Hübner, 2013a). On RR trials, the inhibition of the last response reduces the activation of the correct response which increases the response conflict elicited by bivalent-incongruent stimuli. As a consequence, RR costs are larger for bivalent-incongruent stimuli than for neutral ones.

However, the current experimental condition might represent a special case, because bivalency was uniquely coupled with incongruency. The resulting high perceptual discriminability between the two stimulus types also enabled trial-based strategies, e.g., stimulus-type specific response inhibition. Thus, it is open whether the observed differences in RR costs were exclusively due to amplification or also to stimulus-type specific response inhibition. To test this question, we conducted the next experiment.

## Experiment 2

In this experiment we tried to prevent stimulus-type specific response inhibition. We hypothesized that this might be obtained by also presenting bivalent-congruent stimuli as S2. Because these stimuli are perceptually similar to bivalent-incongruent stimuli (cf. Table 1), participants cannot easily “see” whether a stimulus is incongruent, i.e., whether it poses a high risk of perseveration, or not. Consequently, the strategy to increase response inhibition when a high-risk stimulus is presented should be difficult to apply. Thus, to test whether our hypothesis is valid, we mixed neutral, bivalent-congruent, and bivalent-incongruent S2 in equal proportions.

Assuming that this procedure prevents stimulus-type specific response inhibition (i.e., response inhibition is the same for all stimulus-types), we can formulate the following hypotheses. First, if the pattern of RR costs in Experiment 1 was exclusively due to an automatic ARC by response inhibition (i.e., stimulus-type specific response inhibition was irrelevant in Experiment 1), then we should observe the same results in the present experiment. Second, if the pattern of RR costs in Experiment 1 was exclusively due to stimulus-type (i.e., univalent vs. bivalent) specific response inhibition, then we should find similar RR costs for all stimulus types in the present experiment. Moreover, RR costs for bivalent-incongruent stimuli should be smaller than in Experiment 1. Third, if both ARC and stimulus-type specific response inhibition contributed to the pattern of RR costs in Experiment 1, then we should again find an increase of RR costs for bivalent-incongruent stimuli, but this increase should be smaller than in Experiment 1 (the increase should be reduced by the amount stimulus-type specific response inhibition contributed to the effect in Experiment 1).

Finally, the inclusion of bivalent-congruent stimuli also allowed us to test a prediction of the ARC account (Grzyb and Hübner, 2013a). It follows from this account that RR costs should not be larger for bivalent-congruent stimuli than for neutral ones, because bivalent-congruent stimuli induce no response conflict that could be amplified. Thus, for both bivalent-congruent and neutral stimuli the only factor that is relevant for the size of RR costs is the strength of response inhibition. Because the strength of response inhibition should be the same for both stimulus-types, we expected similar RR costs for neutral and bivalent-congruent stimuli.

### Method

#### Participants

Thirty-six students of the Universität Konstanz participated in the experiment. All participants had normal or corrected-to-normal vision and were either paid 8 Euro per hour or fulfilled a course requirement. Four participants were excluded from analysis, because of poor performance on the task (final sample: 8 males; *M* = 23 years)^1^. Poor performance was defined as RT2 or ER2 larger than two standard deviations above the group mean (RT2 > 1165 ms, ER2 > 18.2%).

#### Stimuli and procedure

In addition to the two stimulus-types in Experiment 1, S2 could also be bivalent-congruent. Similar to bivalent-incongruent stimuli, bivalent-congruent ones consisted of stimulus items of both tasks (a letter *and* a numeral), which, however, both activated the same response. The procedure was identical to Experiment 1 except that neutral, bivalent-congruent, and bivalent-incongruent S2 were presented on one third of the trials, respectively.

### Results

**Figure 3:**
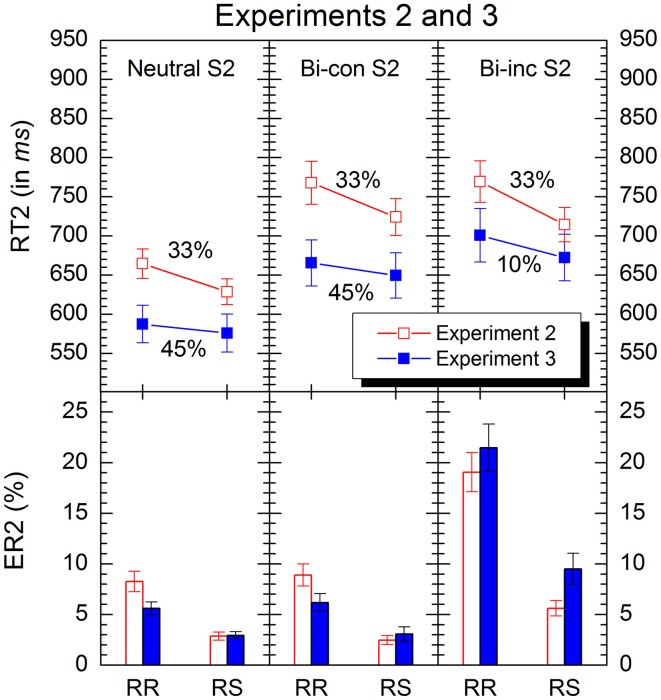
**Mean response times and errors rates in conditions of Experiment 2 (red) and 3 (blue).** “RR” and “RS” denote response repetition and response shifts, respectively. “Bi-con S2” and “bi-inc S2” denote bivalent-congruent and bivalent-incongruent stimuli on Task2, respectively (see Table 1 for details). The percentages indicate the relative proportion of the respective stimulus-types in the experiments. Error bars represent standard errors of the mean.

Trials with RT1 > 1500 ms were not analyzed (2.18% of all trials). Results are depicted in Figure 3.

#### RT1

The mean latency for correct responses to S1 was 603 ms, *SE* = 15.32 ms.

#### ER1

The mean error rate for responses to S1 was 3.97%, *SE* = 0.0052%.

#### RT2

Anticipatory errors (RT2 < 150 ms) and extreme outliers (RT2 > 3500 ms) were excluded from the analysis of the second response (together less than 0.3% in each condition) as well as trials with incorrect responses to S1. Mean latencies of correct responses to S2 were entered into a two-way ANOVA with the independent variables *response transition* (repetition, shift) and *S2 type* (neutral, bivalent-congruent, bivalent-incongruent) realized within participants.

The analysis revealed significant main effects of *S2 type*, *F*(2, 62) = 79.7, *p* < 0.001, and *response transition*, *F*(1, 31) = 26.5, *p* < 0.001. Responses to neutral stimuli were faster than those to bivalent-congruent and bivalent-incongruent ones (*M* = 647 ms, *SE* = 12.58 ms vs. *M* = 746 ms, *SE* = 18.12 ms and *M* = 742 ms, *SE* = 17.53 ms), and RRs were slower than RSs (*M* = 734 ms, *SE* = 14.94 ms vs. *M* = 689 ms, *SE* = 12.71 ms). Concerning the interaction between the two variables, there was only a small trend, *F*(2, 62) = 2.23, *p* = 0.15.

#### ER2

Mean error rates of responses to S2 were subjected to an ANOVA of the same type as for the latencies. The analysis revealed significant main effects of *S2 type*, *F*(2, 62) = 31.7, *p* < 0.001, and *response transition*, *F*(1, 31) = 67.3, *p* < 0.001. There were fewer errors for neutral and bivalent-congruent S2 than for bivalent-incongruent ones (*M* = 5.55%, *SE* = 0.64% and *M* = 5.68%, *SE* = 0.71%, vs. *M* = 12.3%, *SE* = 1.34%), and more errors occurred for RRs than for RSs (*M* = 12.1%, *SE* = 0.95% vs. *M* = 3.65%, *SE* = 0.35%). However, the interaction between the two variables was also significant, *F*(2, 62) = 26.8, *p* < 0.001. Planned comparisons revealed that RR costs for bivalent-incongruent S2 (RR *M* = 19.1%, *SE* = 1.94%, RS *M* = 5.62%, *SE* = 0.76%) were significantly larger than those for neutral ones [RR *M* = 8.24%, *SE* = 1.01%, RS *M* = 2.87%, *SE* = 0.40%; *F*(2, 31) = 30.3, *p* < 0.001] and than those for bivalent-congruent ones [*M* = RR 8.90%, *SE* = 1.10%, RS *M* = 2.47%, *SE* = 0.43%; *F*(2, 31) = 27.5, *p* < 0.001].

### Comparison with experiment 1

We also compared the performance in the present experiment with that in Experiment 1. To this end, we calculated three-way ANOVAs with the independent variable *experiment* (Experiment 1, Experiment 2) realized between-participants and the independent variables *response transition* (repetition, shift) and *S2 type* (neutral, bivalent-incongruent) realized within-participants. We report only significant results involving the between-participant variable *experiment*.

The analyses of RT2 revealed a significant two-way interaction between *experiment* and *S2 type*, *F*(1, 64) = 23.9, *p* < 0.001. The interaction showed that the slowing for responses to bivalent-incongruent compared to neutral S2 was more pronounced in Experiment 1 (neutral *M* = 625 ms, *SE* = 15.72 ms, bivalent-incongruent *M* = 828 ms, *SE* = 27.03 ms) than in Experiment 2 (neutral *M* = 647 ms, *SE* = 12.58 ms, bivalent-incongruent *M* = 742 ms, *SE* = 17.53 ms). The three-way interaction between *experiment*, *S2 type*, and *response transition* was also significant, *F*(1, 64) = 8.46, *p* < 0.01. This reflects the finding that the RR costs for bivalent-incongruent S2 were reliably larger than those for neutral S2 in Experiment 1, and only by trend in the present one. Put differently, whereas RR costs were larger in Experiment 1 compared to Experiment 2 for bivalent-incongruent S2, *F*(1, 64) = 4.48, *p* < 0.05, they did not differ for neutral S2, *F*(1, 64) < 1. In a corresponding analysis of ER2 there were no significant main effects or interactions. Finally, to see whether the basic level of response inhibition differed between the experiments we compared RR costs for neutral stimuli, because they represent a relatively direct measure of response inhibition. This analysis revealed that RR costs in the error rates for neutral stimuli were larger in Experiment 2 than in Experiment 1, *F*(1, 66) = 4.07, *p* < 0.05.

### Discussion

In the latencies, the increase in RR costs between bivalent-incongruent compared to neutral stimuli was again reliable, although, this time, it was significantly smaller than in Experiment 1 (19 vs. 89 ms). In the error rates, the increase in RR costs for bivalent-incongruent stimuli was also reliable, but did not differ between experiments. This pattern of results is in line with our third hypothesis and indicates that in both experiments response inhibition amplified response conflict on RR trials, which increased RR costs. And the fact that RR costs for high-risk stimuli were smaller in the present experiment than in Experiment 1 suggests that some trial-based strategy must also have been effective in our first experiment (significantly increasing RR costs in RT for high-risk stimuli). By including bivalent-congruent stimuli this strategy had little or no effect in the present experiment.

Do our data support the assumption that participants in Experiment 1 had specifically increased response inhibition on-the-fly for high-risk (bivalent-incongruent) stimuli? In our first experiment high-risk stimuli could easily be discriminated perceptually from low-risk stimuli. By including bivalent-congruent stimuli, however, which are low-risk, discriminability was considerably reduced in the present experiment. Consequently, high-risk stimuli could not be detected quickly, which prevented stimulus-type specific response inhibition. Unfortunately, although the assumption of stimulus-type specific response inhibition explains why RR costs were much smaller for bivalent-incongruent stimuli in the present experiment, it cannot account for the fact that the reduction of RR costs occurred only in the latencies. Thus, it seems that some other trial-based strategy was also involved.

A possible additional trial-based strategy in this respect could be to only prepare the upcoming task endogenously if necessary. By including bivalent-congruent stimuli we not only altered stimulus discriminability, but also the proportion of bivalent stimuli. In Experiment 1 only 1/3 of the trials were bivalent, whereas in the present experiment their proportion was 2/3. On bivalent trials the relevant task set has to be selected endogenously on the basis of internal representation (e.g., memory content about the last task). In contrast, on univalent trials the stimulus activates only the correct task set, so that no or only little endogenous control is necessary. Consequently, in univalent contexts participants can reduce their internal control efforts by outsourcing (cf. Mayr and Bryck, 2007) task control to the stimuli.

Thus, because bivalent stimuli were relatively rare in Experiment 1, a favorable trial-based strategy would have been to outsource control, i.e., to rely on stimulus-driven control for task selection if the stimulus is neutral, and to increase top-down control only if a high-risk stimulus was detected. Such a stimulus-dependent task preparation would result in delayed responses to bivalent-incongruent stimuli, because the correct response can only be selected after the relevant task set has endogenously been implemented. Interestingly, delayed responding to bivalent-incongruent stimuli can also explain why RR costs were larger in Experiment 1—simply because response inhibition had more time to bias response selection^2^. The effect of delayed processing on error rates is less clear. On the one hand, more time for response inhibition should also increase RR costs in the error rate. On the other hand, though, accuracy generally increases with response time in flanker-task like paradigms (cf. Hübner et al., 2010). It is difficult to predict how these effects add up. However, it is possible that they cancel each other out, which would explain that the RR costs in the error rates did not differ between our experiments. Thus, stimulus-dependent task preparation might explain the relatively large increase for RR costs for bivalent-incongruent stimuli in the latencies in Experiment 1. We will come back to task preparation in the General Discussion.

Our results clearly indicate that different processing styles were applied in our first two experiments. Was inhibitory control adapted accordingly? The comparison of Experiment 1 and 2 suggests that this was indeed the case. RR costs for neutral stimuli, which represent a relatively direct measure of response inhibition, were larger in Experiment 2 than in Experiment 1. This result indicates that the basic level of response inhibition was larger in Experiment 2, and suggests that overall control strategies (e.g., inhibitory control) were more important in Experiment 2, presumably because trial-based strategies were more difficult to apply.

Another important finding of Experiment 2 is that RR costs were larger for bivalent-incongruent stimuli than for bivalent-congruent ones. This result was predicted by the ARC account. According to this account RR costs were smaller for bivalent-congruent stimuli, because they do not activate the wrong response. Consequently, inhibition and response conflict cannot amplify each other. Our finding is also important for refuting a possible objection. One might have argued that the increased RR costs for bivalent-incongruent stimuli are, at least partly, the result of a scaling effect. Because response times are longer for those stimuli, RR costs also increase. However, mean response times for bivalent-congruent stimuli were similar to those for bivalent-incongruent ones, but RR costs nevertheless differed substantially between these stimulus types. Thus, the increase in RR costs for bivalent-incongruent stimuli is not simply the result of longer response times.

Why was the increase in RR costs for bivalent-incongruent stimuli in Experiment 2 much stronger in accuracy than in the latencies? Notably, an analogous difference holds for the congruency effect, i.e., better performance for bivalent-congruent stimuli compared to bivalent-incongruent ones. The congruency effect was practically absent in response times but substantial in error rates (cf. Figure 3). However, this is not unusual for studies applying compound stimuli (cf. Rogers and Monsell, 1995). Thus, if the effect of incongruency is more pronounced in error rates and if this effect is amplified by response inhibition (ARC) one should expect that the increase in RR costs is also more pronounced in error rates.

Taken together, the results of Experiments 1 and 2 show that, if stimulus-type dependent trial-based strategies are possible, then there is little or no overall strategic control. Moreover, it seems that the summed effects of several processing strategies make it difficult to assess the actual strength of response inhibition. Such effects might also have limited the validity of previous studies (Grzyb and Hübner, 2013a) that were conducted to provide evidence for a strategic adaptation of response inhibition to the overall risk of perseveration. Our present results indicate that applying both bivalent-congruent and bivalent-incongruent stimuli is more appropriate for such an objective.

## Experiment 3

The results of our first two experiments suggest that strategies of adapting overall response inhibition to the risk of perseveration might be applied only if trial-based strategies are prevented, as in the previous experiment. Therefore, we conducted a similar experiment in which the proportion of high-risk stimuli was even further reduced. In Experiment 2, neutral, bivalent-congruent, and bivalent-incongruent stimuli had an equal proportion. In the present experiment, though, bivalent-incongruent stimuli occurred only on 10% of the trials, whereas the other stimulus-types were equal in proportion (45%).

Because the overall risk of response perseveration was rather low (only 10% high-risk, i.e., bivalent-incongruent stimuli), and because trial-based processing was prevented (due to the inclusion of bivalent-congruent stimuli), we expected that the basic level of response inhibition would be adapted to this low risk. As a result, RR costs for neutral and bivalent-congruent stimuli should be substantially smaller than in Experiment 2.

Predicting results for bivalent-incongruent stimuli was more difficult. Because their proportion was rather low, it could be expected that the congruency effect would be relatively large (e.g., Hübner et al., 2010). According to the ARC account (Grzyb and Hübner, 2013a), response inhibition should amplify the negative effects of incongruency only on RR trials thereby increasing RR costs. Thus, it was possible that both effects, i.e., reduced response inhibition and increased effect of incongruency, would counterbalance each other.

### Method

#### Participants

Thirty-four students of the Universität Konstanz participated in the experiment. All participants had normal or corrected-to-normal vision and were either paid 8 Euro per hour or fulfilled a course requirement. Two participants was excluded from analysis because of poor performance on the task (final sample: 9 males; *M* = 23 years), where poor performance was defined as RT2 or ER2 larger than 2 standard deviations above the group mean (RT2 > 1073 ms; ER2 > 13.4%).

#### Stimuli and procedure

The stimuli and procedure were identical to those in Experiment 3 except that, bivalent-incongruent S2 occurred on 11.1% or the trials (8/72), whereas neutral and bivalent-congruent S2 occurred on 44.4% of the trials (32/72), respectively.

### Results

Trials with RT1 > 1500 ms were not analyzed (1.37% of all trials). Results are depicted in Figure 3.

#### RT1

The mean latency for S1 was 541 ms, *SE* = 19.72 ms.

#### ER1

Mean error rate for responses to S1 was 3.12%, *SE* = 0.0032%.

#### RT2

Anticipatory errors (RT2 < 150 ms) and extreme outliers (RT2 > 3500 ms) were excluded from the analysis of second response (together less than 0.3% in each condition) as well as trials with incorrect responses to S1. Mean latencies of correct responses were entered into a two-way ANOVA with the independent variables *response transition* (repetition, shift) and *S2 type* (neutral, bivalent-congruent, bivalent-incongruent) realized within participants.

The analysis revealed significant main effects of *S2 type*, *F*(2, 62) = 45.8, *p* < 0.001, and *response transition*, *F*(1, 31) = 6.30, *p* < 0.05. Responses to neutral stimuli were faster than those to bivalent-congruent (*M* = 582 ms, *SE* = 16.96 ms vs. *M* = 658 ms, *SE* = 20.49 ms; *p* < 0.001) and responses to bivalent-incongruent were slowest (*M* = 687 ms, *SE* = 22.50 ms; *p* < 0.05). Also, RRs were slower than RSs (*M* = 651 ms, *SE* = 17.49 ms vs. *M* = 633 ms, *SE* = 16.43 ms). The interaction between the two independent variables was far from being significant, *F*(2, 62) < 1. More specifically, although RR costs were numerically larger for bivalent-incongruent stimuli (*M* = RR 701 ms, *SE* = 34.03 ms vs. RS *M* = 672 ms, *SE* = 29.78 ms) than for neutral ones (RR *M* = 587 ms, *SE* = 23.97 ms vs. RS *M* = 576 ms, *SE* = 24.34 ms), this difference was not reliable, *F*(1, 31) = 1.21, *p* = 0.28.

#### ER2

Mean error rates for responses to S2 were entered into an ANOVA of the same type as for the latencies. The analysis revealed significant main effects of *S2 type*, *F*(2, 62) = 44.3, *p* < 0.001, and *response transition*, *F*(1, 31) = 28.0, *p* < 0.001. Fewer errors occurred for neutral and bivalent-congruent stimuli than for bivalent-incongruent ones (*M* = 4.27%, *SE* = 0.40% and *M* = 4.62%, *SE* = 0.59%, vs. *M* = 15.5%, *SE* = 1.57%), and RRs produced more errors than RSs (*M* = 11.1%, *SE* = 1.13% vs. *M* = 5.17%, *SE* = 0.65%). The interaction between the two variables was also significant, *F*(2, 62) = 24.1, *p* < 0.001. Planned comparisons revealed that RR costs for bivalent-incongruent S2 (*M* = RR 21.5%, *SE* = 2.33%, RS *M* = 9.51%, *SE* = 1.53%) were larger than those for each of the other two stimulus-types (neutral: RR *M* = 5.61%, *SE* = 0.64%, RS *M* = 2.93%, *SE* = 0.38%; bivalent-congruent: RR *M* = 6.18%, *SE* = 0.88%, *M* = RS 3.07%, *SE* = 0.70%; *p* < 0.001 for each of the two comparisons).

### Comparison with experiment 2

The performance in the present experiment was also compared with that in Experiment 2. We subjected RT2 and ER2 into two separate three-way ANOVAs with the independent variable *experiment* (Experiment 2, Experiment 3) realized between participants and the independent variables *response transition* (repetition, shift) and *S2 type* (neutral, bivalent-congruent, bivalent-incongruent) realized within participants. We report only significant results involving the variable *experiment*.

The analysis of RT2 revealed that participants were faster in Experiment 3 (*M* = 642 ms, *SE* = 12.0 ms) than in Experiment 2 (*M* = 712 ms, *SE* = 9.9 ms), *F*(1, 62) = 3.99, *p* < 0.05. Critically, the interaction between *experiment* and *response transition* was also significant, *F*(1, 62) = 5.23, *p* < 0.05, indicating that RR costs were smaller in the present experiment compared to Experiment 2. Because we specifically expected RR costs to be smaller for neutral and bivalent-congruent S2, we compared the *experiment* × *response transition* interaction for the specific S2 types separately. These further analyses showed that RR costs were significantly smaller in Experiment 3 than in Experiment 2 for neutral S2, *F*(1, 62) = 9.49, *p* < 0.01, and marginally smaller for bivalent-congruent S2, *F*(1, 62) = 3.26, *p* = 0.076. RR costs for bivalent-incongruent S2, however, did not differ significantly between experiments, *F*(1, 62) = 1.81, *p* = 0.18. Finally, we tested whether incongruency had a higher impact due to its low frequency in Experiment 2. The effect of incongruency (bivalent-congruent S2 vs. bivalent-incongruent S2) was larger in Experiment 3 than in Experiment 2, *F*(1, 62) = 6.13, *p* < 0.05.

The error data mirrored the response time data. RR costs were smaller in Experiment 3 than in Experiment 2, yet this interaction did show only as a small trend, *F*(1, 62) = 2.70, *p* = 0.105. Again, we compared the *experiment* × *response transition* interaction for the specific S2 types separately. RR costs were significantly smaller in Experiment 3 than in Experiment 2 for neutral S2, *F*(1, 62) = 5.10, *p* < 0.05, and for bivalent-congruent S2, *F*(1, 62) = 4.96, *p* < 0.05, but not for bivalent-incongruent S2, *F*(1, 62) < 1. Also, the effect of incongruency was larger in Experiment 3 than in Experiment 2, *F*(1, 62) = 4.14, *p* < 0.05.

### Discussion

RR costs were again reliable, and differed between the stimulus types. However, as expected, RR costs for neutral and bivalent-congruent stimuli were significantly smaller than in Experiment 2. This result supports our hypothesis that the basic level of response inhibition is strategically controlled, if trial-based strategies cannot be applied. Compared to Experiment 2, the smaller proportion of high-risk stimuli in the present experiment reduced the risk of perseveration errors. Consequently, response inhibition was generally smaller.

For bivalent-incongruent stimuli, the smaller response inhibition did not lead to smaller RR costs. This confirms the idea that the size of RR costs for bivalent-incongruent stimuli depends on at least two factors; the strength of response inhibition and the magnitude of the response conflict, the latter passively increasing RR cost (ARC). While response inhibition was reduced in the present experiment, response conflict was larger, which can be seen in a larger congruency effect even on RS trials (cf. Figure 3). Therefore, the finding of similar RR costs for bivalent-incongruent stimuli in Experiments 2 and 3 is in line with our assumption that the effect of reduced overall inhibition on the size of RR costs was compensated for by the larger amplification effect due to the increased response conflict on trials with bivalent-incongruent stimuli (Grzyb and Hübner, 2013a).

## General discussion

The present study investigated to what extent response inhibition can strategically be adjusted to the overall demands of a task context. According to the response-inhibition account of RR effects in task switching (Hübner and Druey, 2006; see also Marí-Beffa et al., 2012), responses are strategically inhibited to control the error rate in task-switching contexts, where perseveration errors are likely to occur due to residual activations left over from previous task performance. Because the risk of committing such errors is relatively high for bivalent-incongruent stimuli, conditions with a high proportion of these stimuli pose a higher overall risk of perseveration errors than conditions with a small proportion. Therefore, it is likely that individuals strategically increase the basic level of response inhibition under such conditions. In a previous study, however, no such adaptation effect was found (Grzyb and Hübner, 2013a). Although RR costs were larger for bivalent-incongruent stimuli than for neutral ones, this effect was independent of their proportion. However, in Grzyb and Hübner (2013a) study, low- and high-risk stimuli could easily be discriminated perceptually. Thus, instead of an overall inhibition strategy, a trial-based strategy could have been applied. For instance, response inhibition could have been increased on-the-fly after a high-risk stimulus was detected.

To test the strategic-adaptation hypothesis more strictly, we therefore had to establish a condition in which trial-based strategies are hard to apply. This was realized in Experiment 2 by also presenting bivalent-congruent stimuli in addition to neutral and bivalent-incongruent ones. Bivalent-congruent stimuli also pose a low risk of response perseveration, but are difficult to discriminate perceptually from bivalent-incongruent stimuli. Accordingly, trial-based strategies should be hard to apply with this mixture of stimulus types. For comparison, however, we first (Experiment 1) collected data in a similar way as Grzyb and Hübner (2013a). Indeed, comparing the results of our first two experiments revealed that the difference in RR costs between bivalent-incongruent and neutral stimuli was smaller in Experiment 2. This result indicates that some trial-based strategy is applied if high- and low-risk stimuli can easily be discriminated and that this strategy further increase RR costs for bivalent-incongruent stimuli.

Importantly, RR costs for neutral stimuli were larger in Experiment 2, compared to Experiment 1. Because these cost can be considered as a relatively pure measure of response inhibition (e.g., Grzyb and Hübner, 2013a), this result shows that the basic level of response inhibition was generally larger in Experiment 2. This finding supports our idea that the basic level of inhibition is strategically adapted, given that trail-based strategies cannot be applied. If our idea holds, then the proportion of high-risk stimuli should have an effect on RR costs in conditions where stimulus types are mixed as in Experiment 2. This hypothesis was tested in Experiment 3. In comparison to Experiment 2, we reduced the proportion of bivalent-incongruent stimuli by 70%. As a result, this reduction caused smaller RR costs, which strongly supports the strategic-adaptation hypothesis of response inhibition.

Previous studies yielded only indirect evidence for strategic adaptation of response inhibition to the risk of response perseveration errors. After comparing the effects in pure and mixed task contexts (where only one or several tasks are performed, respectively), several authors argued for an *all-or-none adaptation* of response inhibition and suggested that the last response might be inhibited only in mixed but not in pure task contexts (Steinhauser et al., 2009; Marí-Beffa et al., 2012). Our study extends this view by demonstrating a *gradual adaptation* of response inhibition in mixed contexts to the overall risk of response perseveration errors.

The comparison of Experiments 1 and 2 suggests that some trial-based strategy was applied in Experiment 1. One possible strategy seems to be that participants increased response inhibition on-the-fly when a high-risk stimulus was detected. The stronger response inhibition should affect RR costs for high-risk stimuli in both response times and error rates. However, we merely observed effects on RR costs in the latencies and not in the error rates. Therefore, we concluded that a different trial-based strategy must have been applied. Because two thirds of the stimuli in Experiment 1 were neutral, exogenous activation was largely sufficient to select the correct task and response on the corresponding trials. Only on trials with bivalent stimuli the task had to be selected endogenously. Moreover, the different stimulus types could easily be discriminated. Therefore, a possible strategy was to prepare the required task only if necessary, i.e., when a bivalent-incongruent stimulus or conflict was detected. Such a strategy presumably minimized mental effort by outsourcing task control (cf. Mayr and Bryck, 2007). Its drawback, however, was that on bivalent-incongruent trials, the task had to be selected after stimulus onset, which increased the response time and interference (e.g., Rogers and Monsell, 1995; Steinhauser and Hübner, 2007). If we assume that the effects of response inhibition increase with stimulus processing time, then such a stimulus-type dependent task preparation also explains why RR costs in the response times for bivalent-incongruent stimuli were larger in Experiment 1 than in Experiment 2. In the error rates, there was no difference in RR costs between the experiments, because the effect of the increased response inhibition was presumably counterbalanced by the fact that accuracy generally increases with response time (e.g., Hübner et al., 2010).

### Implication for alternative accounts of RR costs

The present results are also relevant with respect to other accounts of RR costs in task-switching. For example, one class of accounts explains RR costs in task switching as a result of binding and strengthening. According to this idea (Meiran, 2000), a category-response (C-R) rule is strengthened after a response was selected by this rule, whereas other rules associated with the same response are weakened. As a consequence, if the task switches and the same response needs to be selected, it has to be activated by the just weakened rule, which explains the costs (see also Schuch and Koch, 2004).

Closely related is the idea that partial matches between the previous and the current processing episode lead to interference with current processing, because the previous episode is automatically retrieved if any of its features repeats (Altmann, 2011). On task-switch trials, where the response switches, there is no overlap between the previous and the current episode and, therefore, no interference. In contrast, if the response repeats then some episodic features (i.e., the response) overlap between the episodes. Hence, the pervious episode is retrieved eliciting interference with current processing which worsens performance.

These alternative accounts share the common assumption that RR costs are caused exclusively by non-strategic, bottom-up mechanisms. As a consequence, they have difficulties in explaining a modulation of RR costs by the proportion of high-risk stimuli. The response inhibition account, in contrast, explains this context effect with the strategic inhibition of the last response in order to prevent response perseveration errors. Thus, the proportion effect observed in the present study strongly suggest that, even if binding and retrieval mechanisms may partly account for RR effects in task-switching, an additional mechanism that can be controlled strategically, has to be assumed. An obvious candidate in this respect is response inhibition (cf. Marí-Beffa et al., 2012; Grzyb and Hübner, 2013a).

## Conclusion

The present study supports the idea that the strength of response inhibition can strategically be adapted to the overall risk of perseveration errors, e.g., to the proportion of high-risk stimuli. However, such a strategy is mainly applied when trial-based strategies are not feasible, for instance, because low- and high-risk stimuli are difficult to discriminate.

### Conflict of interest statement

The authors declare that the research was conducted in the absence of any commercial or financial relationships that could be construed as a potential conflict of interest.

## Footnotes

^1^The exclusion of participants in this and in the following experiment did not change the pattern of results nor the conclusion of the study.

^2^In fact, from unpublished analyses we know that RR costs in latencies generally increase with response time.
